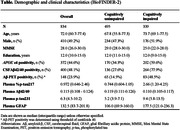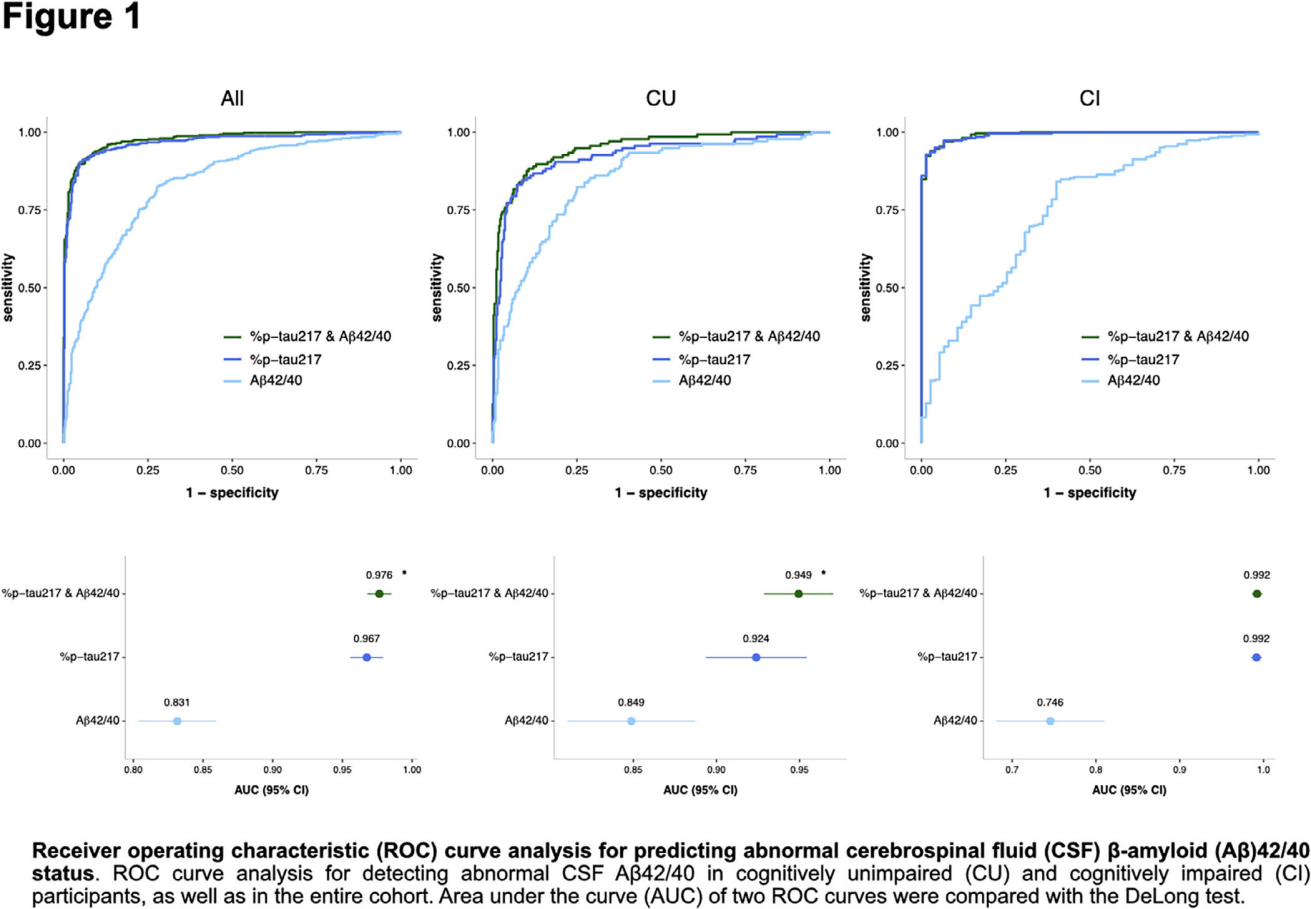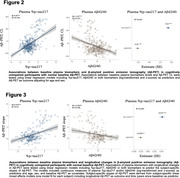# A combination of plasma phospho‐tau217 and Aβ42/40 detects early development of amyloid pathology in cognitively unimpaired individuals

**DOI:** 10.1002/alz.092307

**Published:** 2025-01-09

**Authors:** Shorena Janelidze, Nicolas R. Barthélemy, Gemma Salvadó, Sebastian Palmqvist, Niklas Mattsson‐Carlgren, Vitaliy Ovod, James G. Bollinger, Yingxin He, Suzanne E. Schindler, Kaj Blennow, Erik Stomrud, Randall J. Bateman, Oskar Hansson

**Affiliations:** ^1^ Clinical Memory Research Unit, Department of Clinical Sciences, Lund University, Malmö Sweden; ^2^ Washington University School of Medicine, St. Louis, MO USA; ^3^ Clinical Memory Research Unit, Lund University, Lund Sweden; ^4^ Clinical Memory Research Unit, Department of Clinical Sciences Malmö, Faculty of Medicine, Lund University, Lund, Sweden, Malmö Sweden; ^5^ Clinical Memory Research Unit, Department of Clinical Sciences Malmö, Faculty of Medicine, Lund University, Lund Sweden; ^6^ Washington University in St. Louis School of Medicine, St. Louis, MO USA; ^7^ Department of Psychiatry and Neurochemistry, Institute of Neuroscience and Physiology, University of Gothenburg, Mölndal Sweden; ^8^ Memory Clinic, Skåne University Hospital, Malmö Sweden; ^9^ Clinical Memory Research Unit, Department of Clinical Sciences, Lund University, and Memory Clinic, Skåne University Hospital, Malmö Sweden

## Abstract

**Background:**

Phase 3 trials of successful anti‐amyloid therapies in Alzheimer's disease (AD) have indicated better clinical efficacy in people with less severe disease. Plasma biomarkers will be essential for efficient screening of participants in future primary prevention clinical trials testing anti‐amyloid therapies in cognitively unimpaired (CU) people with normal brain β‐amyloid (Aβ) levels by PET who are at high risk of accumulating Aβ. Here we investigated if combining plasma phospho‐tau (p‐tau)217 and Aβ42/40 could be useful to predict subsequent development of Aβ pathology in CU with normal brain Aβ by PET at baseline.

**Method:**

We included 495 CU individuals and 339 patients with cognitive impairment (CI) from the BioFINDER‐2 study (Table). Plasma Aβ42/40 (mass spectrometry), the ratio of p‐tau217 to non‐phosphorylated tau (%p‐tau217; mass spectrometry), p‐tau231 and GFAP (immuno‐assays) were measured at baseline. Among CU participants, 224 underwent longitudinal Aβ‐PET assessments. Threshold of 40 centiloids and previously established CSF Aβ42/40 cut‐offs were applied to define Aβ‐PET and CSF Aβ status, respectively. For replication we included 249 CU individuals from BioFINDER‐1.

**Result:**

When detecting abnormal CSF Aβ‐status, a combination of plasma %p‐tau217 and Aβ42/40 showed better performance than individual biomarkers in CU (AUC_range_=0.949, p_diff_<0.024) but not in CI participants (Figure 1). Further, in CU participants with normal baseline Aβ‐PET, baseline plasma %p‐tau217 and Aβ42/40 were associated with continuous baseline Aβ‐PET measures (Figure 2) and increases in Aβ‐PET signal over time (Figure 3). Associations of plasma %p‐tau217 and Aβ42/40 with baseline Aβ‐PET (%p‐tau217: β=4.23, p<0.001; Aβ42/40: β=‐2.61, p<0.001) and longitudinal Aβ‐PET (%p‐tau217: β=0.527, p<0.001; Aβ42/40: β=‐0.259, p=0.002) were also significant in the models combining the two baseline biomarkers as predictors (Figures 2‐3). Similarly, in BioFINDER‐1, baseline plasma p‐tau217 (immuno‐assay) and Aβ42/40 were independently associated with longitudinal changes in CSF Aβ42/40 in CU participants with normal CSF Aβ42/40 at baseline. Plasma p‐tau231 and GFAP did not provide any independent value when combined with %p‐tau217and Aβ42/40.

**Conclusion:**

Combining plasma measures of p‐tau217 and Aβ42/40 could be useful for predicting development of Aβ pathology in people with early stages of subthreshold Aβ accumulation. These biomarkers might thus facilitate screening of participants for future primary prevention trials.